# Supplementary figures and images for: Systematic single cell RNA sequencing analysis reveals unique transcriptional regulatory networks of Atoh1-mediated hair cell conversion in adult mouse cochleae
Source: PLoS One. 2023 Dec 11;18(12):e0284685. doi: 10.1371/journal.pone.0284685 (PMC10712870; doi:10.1371/journal.pone.0284685)

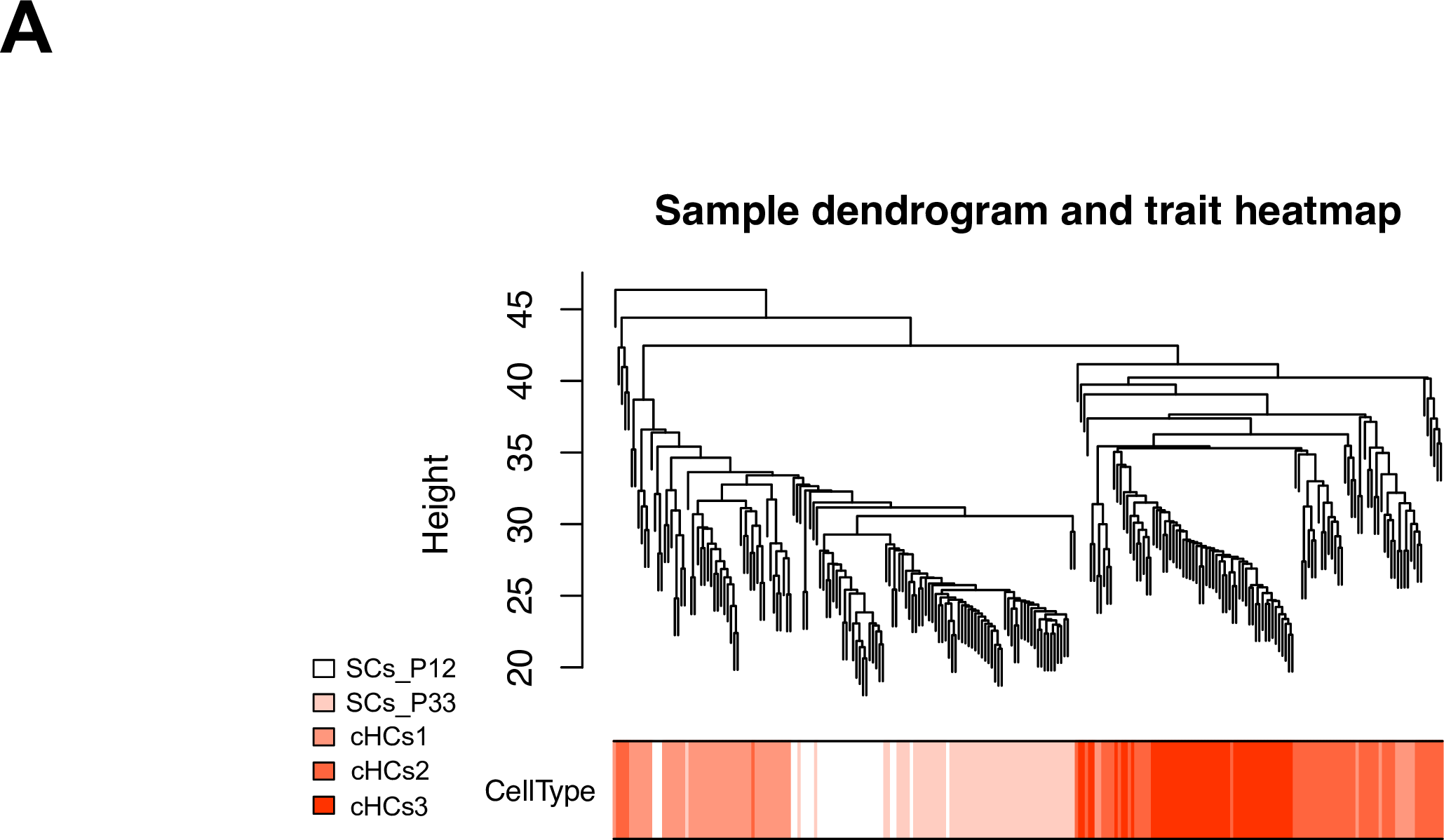

Supplement: S1 Fig — (A) Sample clustering was conducted to detect outliers. This analysis was based on the expression data of top 4000 variable genes in the total 251 cells with 161 Atoh1-HA+ cHCs and 90 SCs available in Yamashita et al [7] (S2 Table). Genes were clustered based on a dissimilarity measure. The branches correspond to modules of highly interconnected groups of genes. Colors in the horizontal bar represent the five cell types assigned by scRNA-seq analysis. (B) Selection of the soft-thresholding powers for scale-free co-expression network. The left panel showed the scale-free fit index versus soft-thresholding power. The right panel displayed the mean connectivity versus soft-thresholding power. Power 4 was used. (ZIP) [file pone.0284685.s001.zip › S1 A.tif]

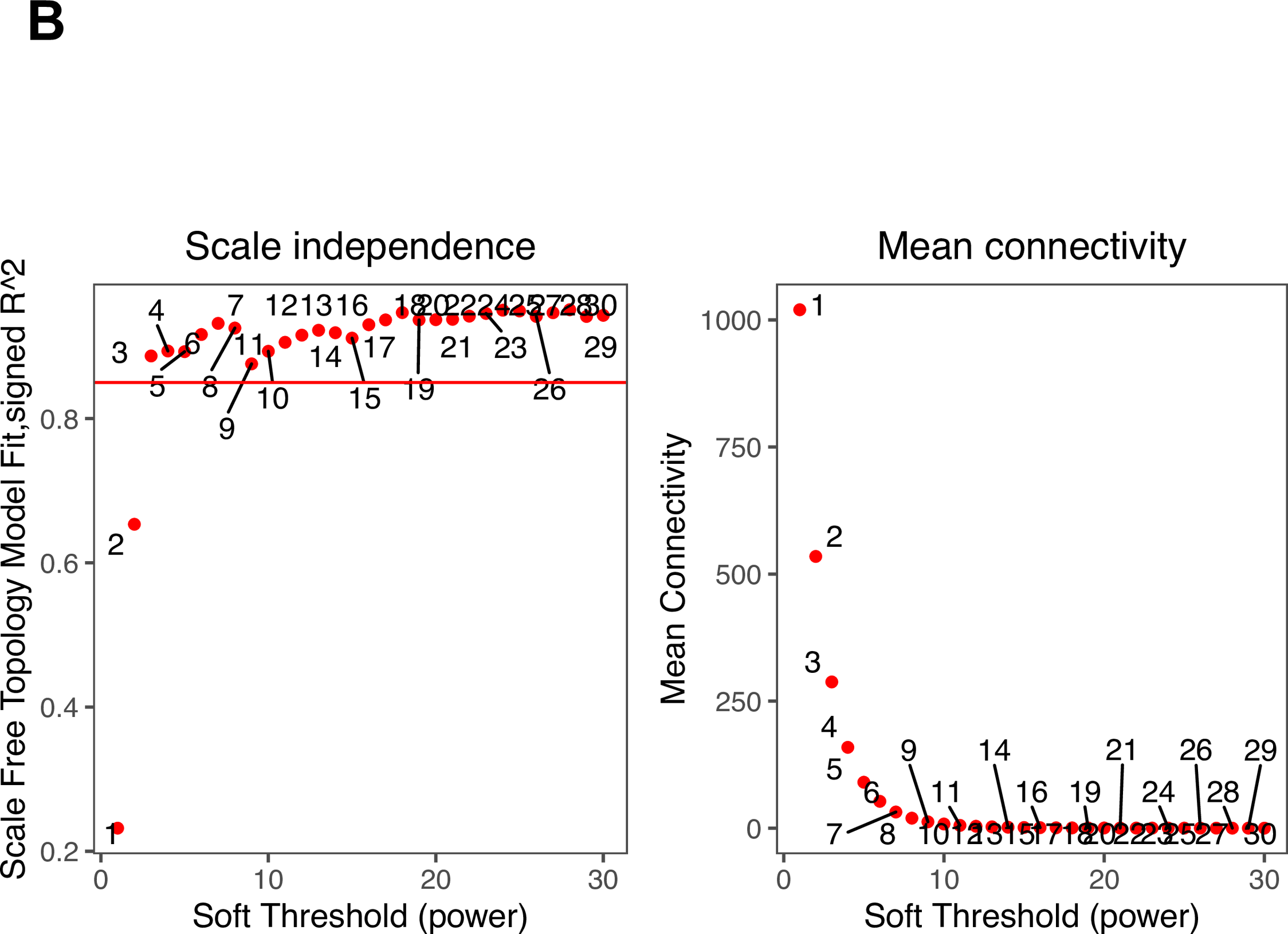

Supplement: S1 Fig — (A) Sample clustering was conducted to detect outliers. This analysis was based on the expression data of top 4000 variable genes in the total 251 cells with 161 Atoh1-HA+ cHCs and 90 SCs available in Yamashita et al [7] (S2 Table). Genes were clustered based on a dissimilarity measure. The branches correspond to modules of highly interconnected groups of genes. Colors in the horizontal bar represent the five cell types assigned by scRNA-seq analysis. (B) Selection of the soft-thresholding powers for scale-free co-expression network. The left panel showed the scale-free fit index versus soft-thresholding power. The right panel displayed the mean connectivity versus soft-thresholding power. Power 4 was used. (ZIP) [file pone.0284685.s001.zip › S1 B.tif]

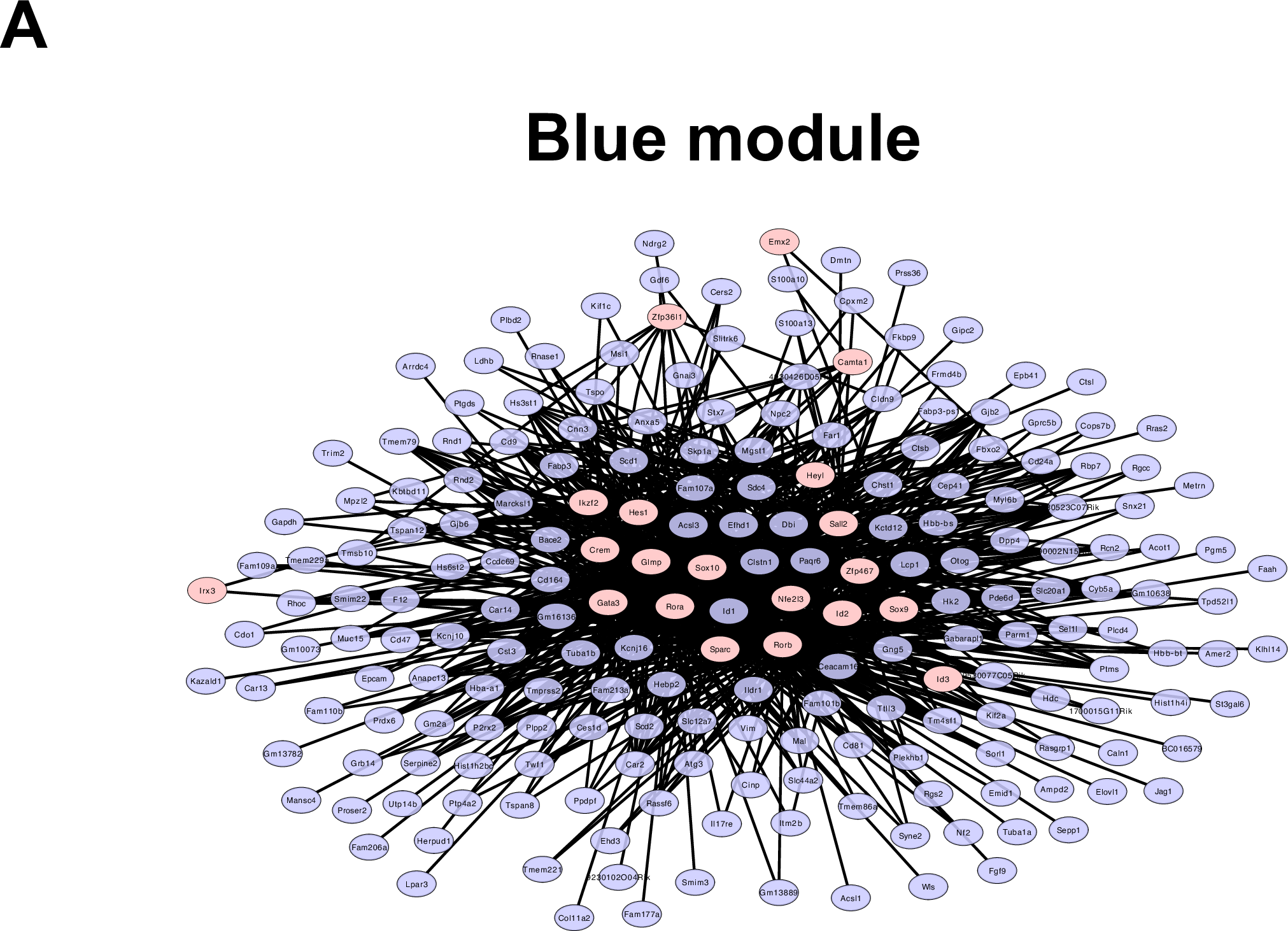

Supplement: S2 Fig — (A-D) The network was derived using the ARACNE program and constructed using Cytoscape. The key regulators (in red) are connected with their targets (in purple). (ZIP) [file pone.0284685.s002.zip › S2 A.tif]

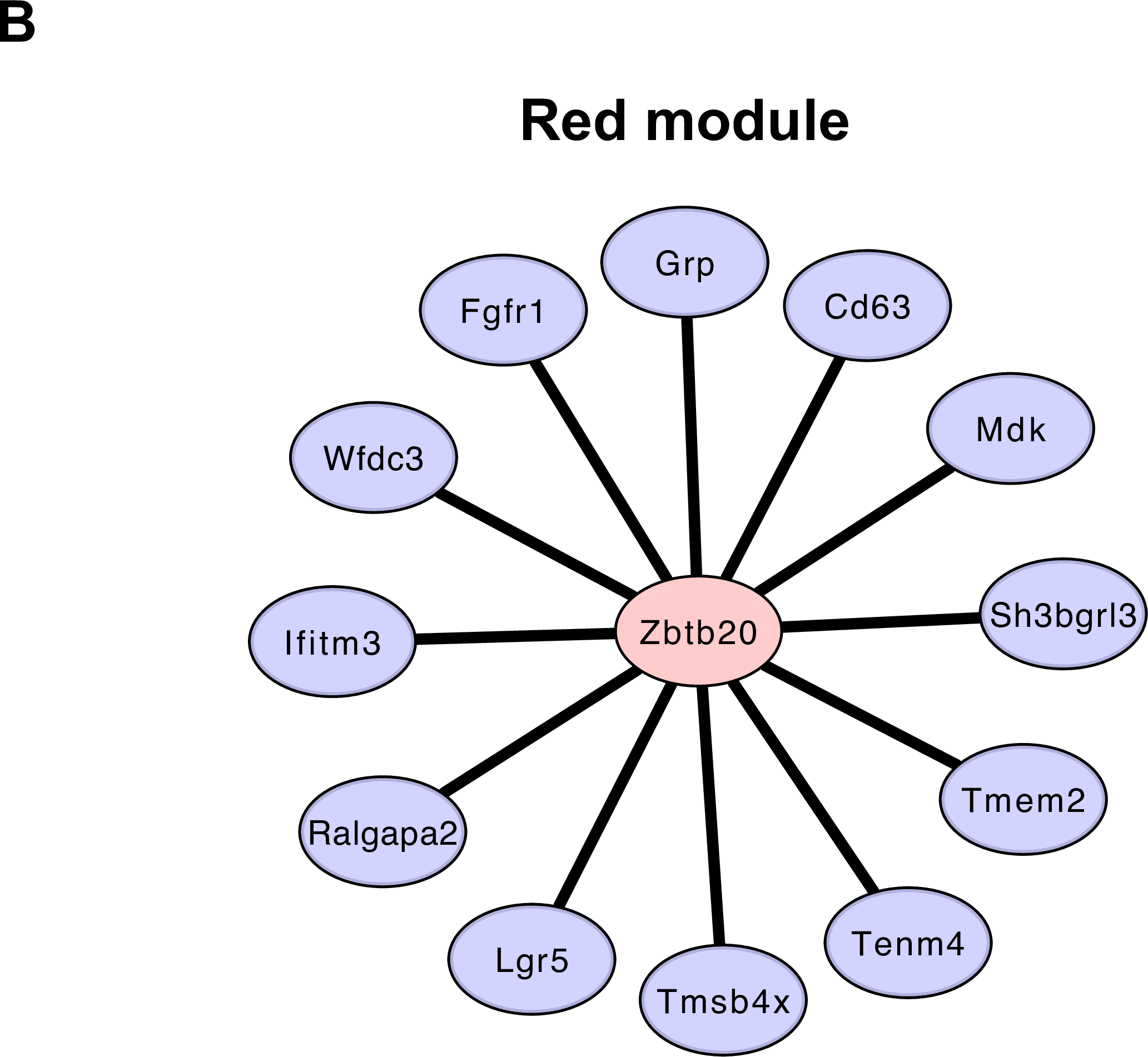

Supplement: S2 Fig — (A-D) The network was derived using the ARACNE program and constructed using Cytoscape. The key regulators (in red) are connected with their targets (in purple). (ZIP) [file pone.0284685.s002.zip › S2 B.tif]

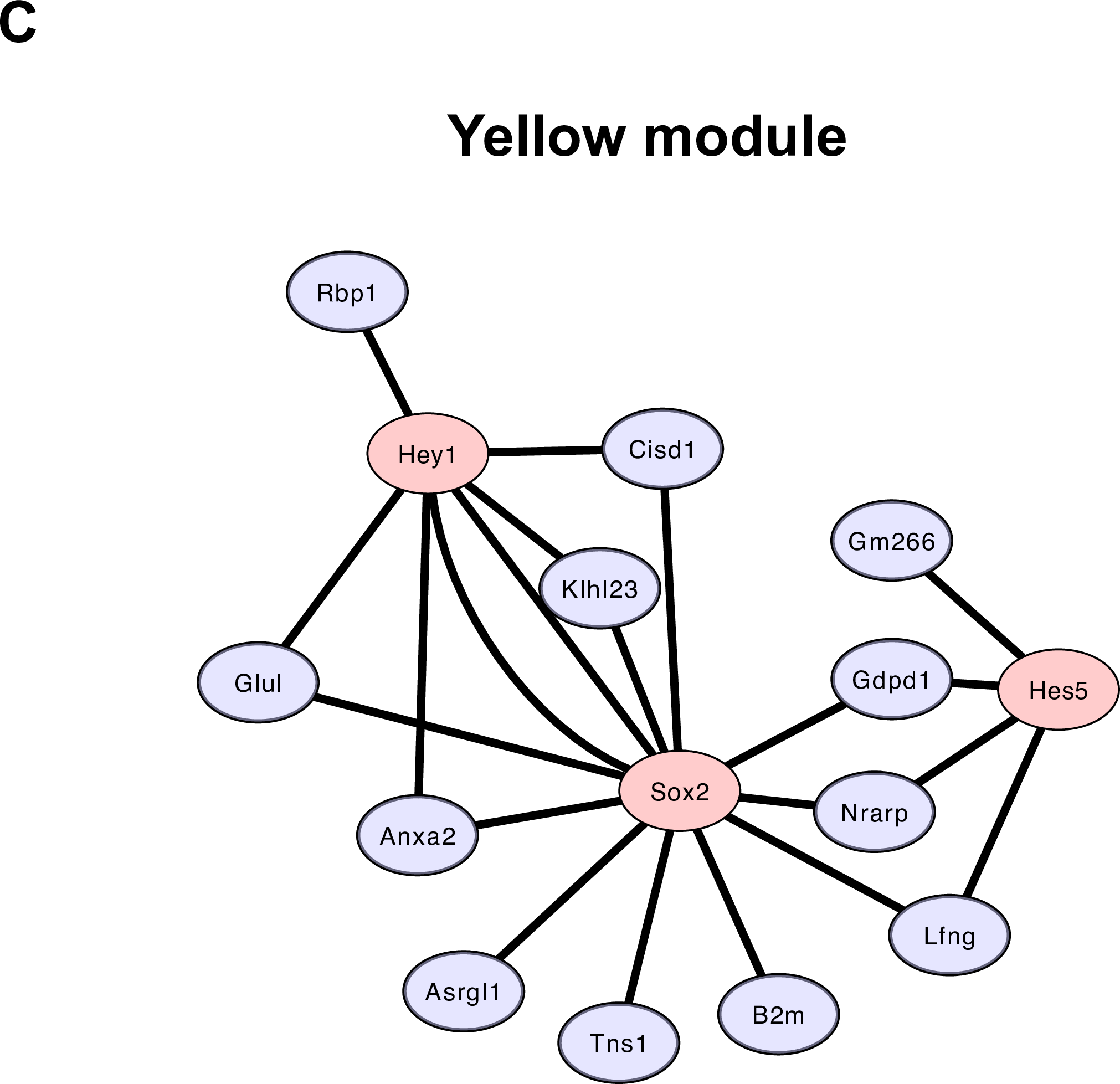

Supplement: S2 Fig — (A-D) The network was derived using the ARACNE program and constructed using Cytoscape. The key regulators (in red) are connected with their targets (in purple). (ZIP) [file pone.0284685.s002.zip › S2 C.tif]

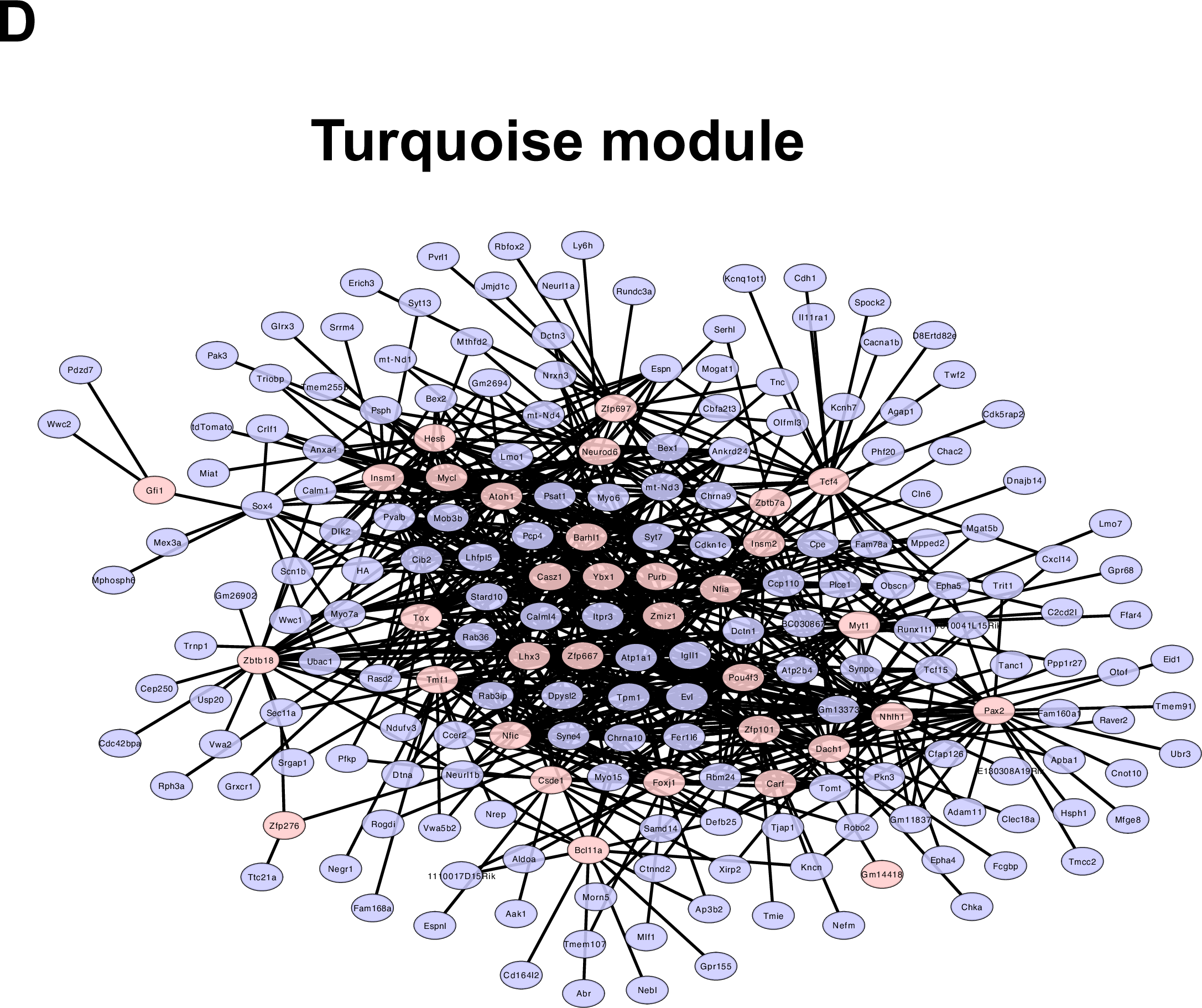

Supplement: S2 Fig — (A-D) The network was derived using the ARACNE program and constructed using Cytoscape. The key regulators (in red) are connected with their targets (in purple). (ZIP) [file pone.0284685.s002.zip › S2 D.tif]

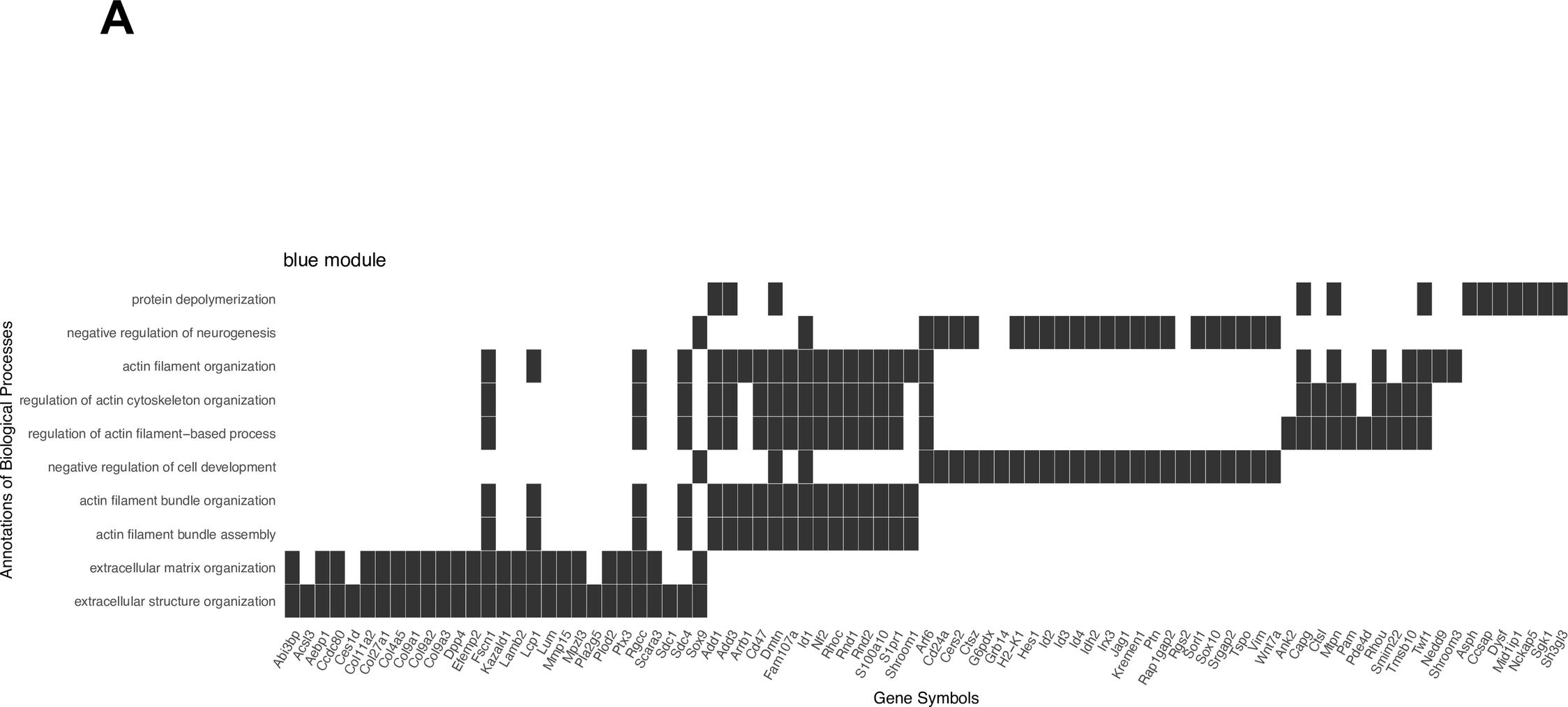

Supplement: S3 Fig — (A) Gene ontology (GO) analysis of genes in the blue module (SC—cHC1) showed enrichment of biological processes related to early-stage hair cell development, such as Notch signaling pathway, regulation of mechanoreceptor differentiation, and epithelial proliferation (B) GO analysis of the red module (cHC1—cHC2) revealed pathways related to organ growth and morphogenesis, cell and tissue migration, indicating the dynamic regulation of conversion-associated genes in this middle stage. (C) GO analysis of the yellow module (cHC1—cHC2) showed enrichment of genes associated with similar processes as the red module, supporting their potential role in the middle stage of hair cell conversion. (D) GO analysis of the turquoise module (cHC2 –cHC3) suggested pathways such as synapse organization and neuroepithelial cell differentiation, indicating their potential role in the later stages of hair cell conversion. (ZIP) [file pone.0284685.s003.zip › S3 A.tif]

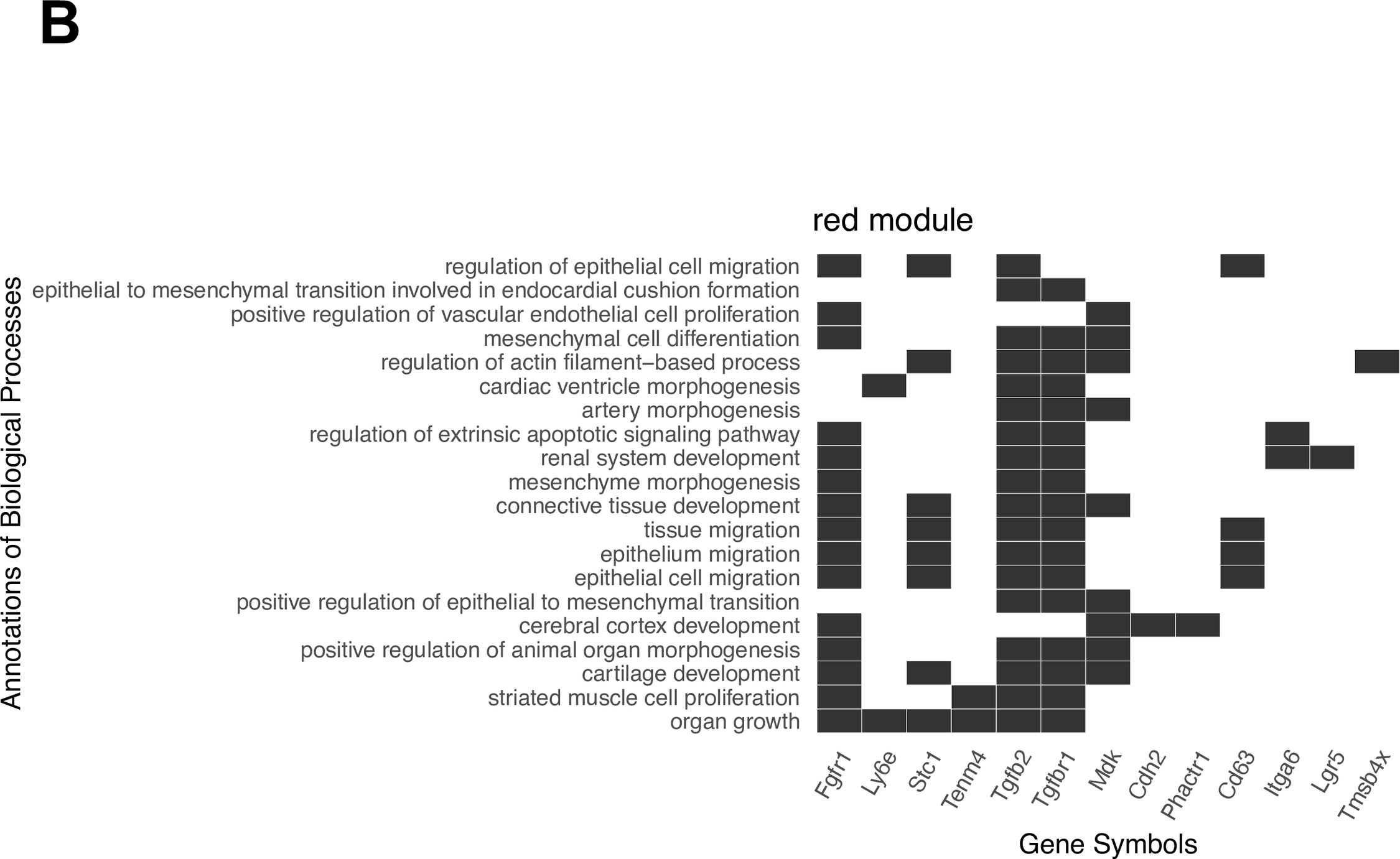

Supplement: S3 Fig — (A) Gene ontology (GO) analysis of genes in the blue module (SC—cHC1) showed enrichment of biological processes related to early-stage hair cell development, such as Notch signaling pathway, regulation of mechanoreceptor differentiation, and epithelial proliferation (B) GO analysis of the red module (cHC1—cHC2) revealed pathways related to organ growth and morphogenesis, cell and tissue migration, indicating the dynamic regulation of conversion-associated genes in this middle stage. (C) GO analysis of the yellow module (cHC1—cHC2) showed enrichment of genes associated with similar processes as the red module, supporting their potential role in the middle stage of hair cell conversion. (D) GO analysis of the turquoise module (cHC2 –cHC3) suggested pathways such as synapse organization and neuroepithelial cell differentiation, indicating their potential role in the later stages of hair cell conversion. (ZIP) [file pone.0284685.s003.zip › S3 B.tif]

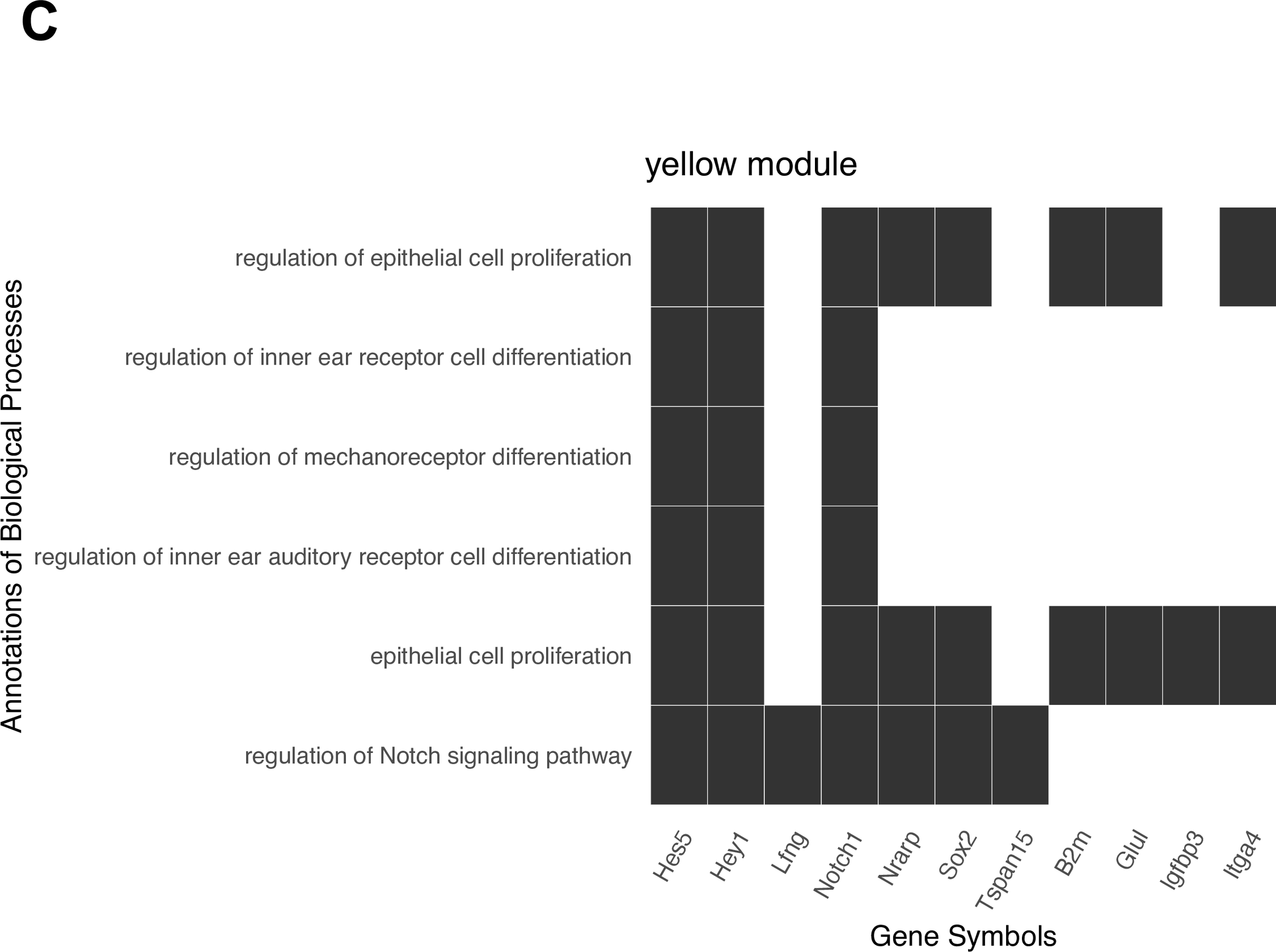

Supplement: S3 Fig — (A) Gene ontology (GO) analysis of genes in the blue module (SC—cHC1) showed enrichment of biological processes related to early-stage hair cell development, such as Notch signaling pathway, regulation of mechanoreceptor differentiation, and epithelial proliferation (B) GO analysis of the red module (cHC1—cHC2) revealed pathways related to organ growth and morphogenesis, cell and tissue migration, indicating the dynamic regulation of conversion-associated genes in this middle stage. (C) GO analysis of the yellow module (cHC1—cHC2) showed enrichment of genes associated with similar processes as the red module, supporting their potential role in the middle stage of hair cell conversion. (D) GO analysis of the turquoise module (cHC2 –cHC3) suggested pathways such as synapse organization and neuroepithelial cell differentiation, indicating their potential role in the later stages of hair cell conversion. (ZIP) [file pone.0284685.s003.zip › S3 C.tif]

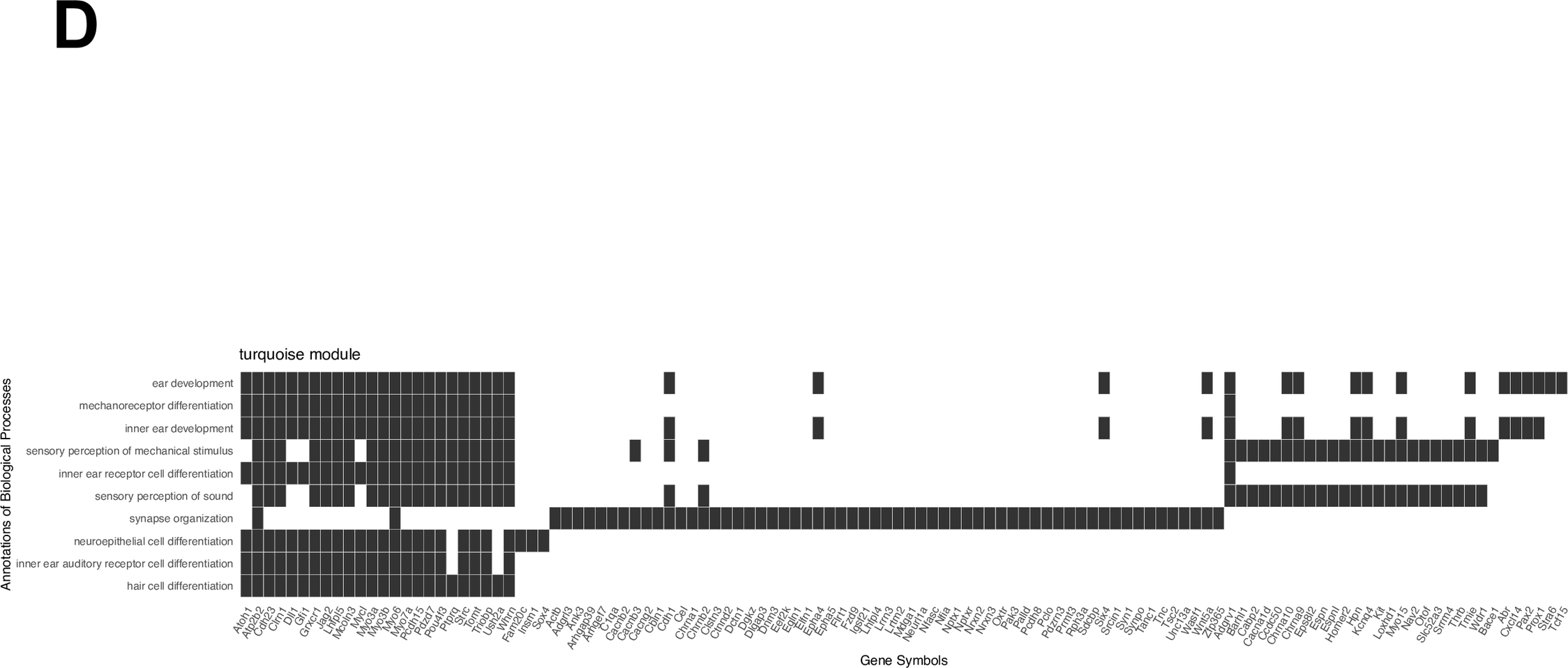

Supplement: S3 Fig — (A) Gene ontology (GO) analysis of genes in the blue module (SC—cHC1) showed enrichment of biological processes related to early-stage hair cell development, such as Notch signaling pathway, regulation of mechanoreceptor differentiation, and epithelial proliferation (B) GO analysis of the red module (cHC1—cHC2) revealed pathways related to organ growth and morphogenesis, cell and tissue migration, indicating the dynamic regulation of conversion-associated genes in this middle stage. (C) GO analysis of the yellow module (cHC1—cHC2) showed enrichment of genes associated with similar processes as the red module, supporting their potential role in the middle stage of hair cell conversion. (D) GO analysis of the turquoise module (cHC2 –cHC3) suggested pathways such as synapse organization and neuroepithelial cell differentiation, indicating their potential role in the later stages of hair cell conversion. (ZIP) [file pone.0284685.s003.zip › S3 D.tif]

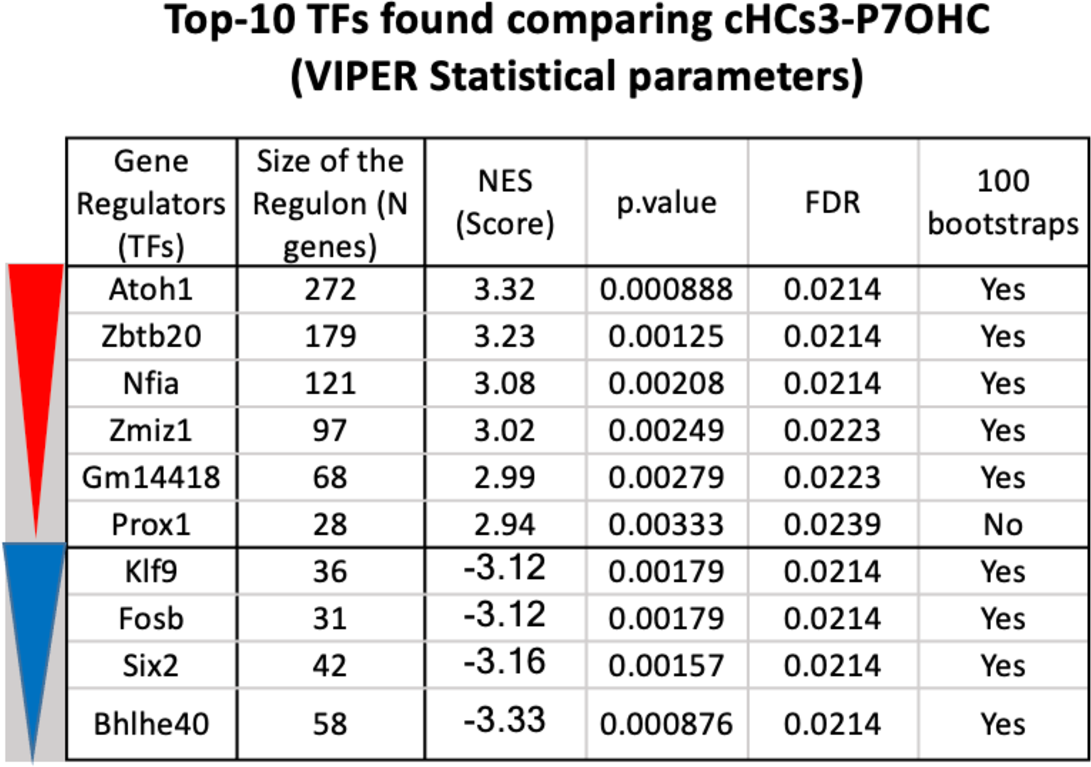

Supplement: S4 Fig — Table showing the top 10 up- and down-regulated transcription factors found by VIPER with corresponding parameters (normalized enrichment score (NES), p-value, false discovery rate (FDR), and bootstraps). (TIF) [file pone.0284685.s004.tif]
