# Supplementary material for: Systematic single cell RNA sequencing analysis reveals unique transcriptional regulatory networks of Atoh1-mediated hair cell conversion in adult mouse cochleae
Source: PLoS One. 2023 Dec 11;18(12):e0284685. doi: 10.1371/journal.pone.0284685 (PMC10712870; doi:10.1371/journal.pone.0284685)
Supplement: Attachment — Submitted filename: PONE-D-22-20296_Reviewer comments.pdf [file pone.0284685.s008.pdf]

SPECIFIC POINTS

1. The term ‘master regulator’ appears to be used quite loosely throughout the paper and is hence misleading. For example, in lines 333-336, the authors use this terminology to refer to several differentially expressed transcription factors, whose functions in SC-to-HC fate conversion have not even been studied to date (by their own admission). What makes them ‘master regulators’ as opposed to just ‘transcriptional regulators’? In addition, in lines 92-93, the authors state that master regulators “…are defined as transcription factors that differentially regulate sets of target genes (regulons).” This is not a correct definition of MRs. The authors are describing a regulatory molecule, not a master regulator. Here’s an example of a study that discusses and makes recommendations on this topic: https://www.ncbi.nlm.nih.gov/pmc/articles/PMC3718559/
The authors should instead use ‘regulators’, or ‘transcriptional regulators’ or ‘transcription factors’ where applicable throughout their manuscript.

Answer: This is a real good suggestion. We have made changes to all “master regulator” to either “Transcription factors” or “regulators” to avoid confusion to the readers.

This is an acceptable resolution, but please check carefully again. I found at least 4 instances where ‘master’ is still in use: lines 21, 92, 284 and 450 of the revised manuscript. In line 450, deleting ‘master’ is not going to be sufficient because you’ll be left with an odd title “Detection of Regulators in the Regulatory Networks”.

2. It is well known that distance in the UMAP space doesn’t necessarily indicate transcriptional similarity. The authors even acknowledge this point explicitly in lines 227-229. Yet, they make the claim that cHC3 and OHC_P1 have similar transcriptomes because of cluster proximity in lines 219-222. This should be corrected. The authors are however not wrong in suggesting that cHC3s appear more similar to OHC_P1 than older OHCs; support for this is clearer in the correlation matrix in Figure 3C and the heatmap in Figure 3B.

Answer: We made correction on that point.

This reviewer appreciates the authors’ efforts but this sentence is worded poorly to the point of being confusing and even misleading. These are the phrases they need to change: “devoted to”, “development tract”, “specified to”. In addition, the trajectory doesn’t link OPC_P1 to PC_7 in Figure 3A, so the authors are advised not to claim that OPC P1 mature to DC/PC at P7.

3. Correction necessary in lines 229-230: A heatmap (of top DEGs) doesn’t validate a UMAP.

Answer: We made correction on that point.

The sentence in Line 242 beginning in “We also analyzed’ is no longer necessary because of the way the subsequent sentence is framed. That resolves the comment I previously made. However, they made another change in the related sentence that comes right after it that makes no sense. The sentence reads “Heatmap of the top 10 DEGs for each cluster clearly showed that during the dynamic change from cHC1-3, a panel of genes were regulated…” How can they know the panel of genes were regulated simply based on a heatmap? The sentence needs to be rephrased.

4. In lines 237-240, the authors suggest that genes that were highly expressed in cHC1-3 but showed limited expression in OHC_P1 could be downstream targets of Atoh1. This explanation doesn’t make sense: Why would Atoh1-regulated targets be selectively expressed in cHC1-3 when OHC_P1 also express Atoh1?

Answer: We explained in Line 425-427: “Moreover, additional genes which are not expressed in nature HC development are also induced during Atoh1-mediated conversion which are likely due to persistent Atoh1 ectopic expression, pervious SC fates, and/or the need for additional transcription factors.”

In order to avoid confusion, we modified in Line 320-322: “Genes that were highly expressed in cHC1-3 but had limited expression in wild-type OHC_P1 could be either direct or indirect downstream effectors of persistent ectopic Atoh1 expression, pervious SC fates, and/or the need for additional transcription factors.”

This is an acceptable change but please replace ‘either direct or indirect downstream effectors of’ with ‘due to’.

5. In lines 243-245, the authors suggest that their results in Figures S4B-S4C suggest that maturation stages of cHC1-3 are “similar to the natural HC development”. Those plots do show trajectories across different cell stages/types but cannot be used to make inferences regarding similarity of developmental progression. The heatmap in Figure 3B does provide some support to this statement though, so the authors are advised to reconsider the way they frame this point.

It is also expected to see two bifurcation in Figure S4B-C as SC to cHCs conversion are different from nature HC development. Therefore, we modified the Line 325-327 to: “Addition of cHCs to the cell trajectory shows bifurcation branches of cHCs and nature HC development (Figure S3A and B)” as the original statement is inappropriate.

This is in line 255, not 325. In addition, this sentence indicating addition of cHCs results in bifurcation is simply stating an observation. The authors should also write what they infer from it. Also the word ‘nature’ needs to be changed.

6. Panels in Figure 4 are highly redundant. Most if not all of Figure 4B should be supplemental; anything remaining could be merged with Figure 4A. For example, size of the regulon could be useful in evaluating the data in Figure 4A, so those values could be directly indicated in Figure 4A and the rest of Figure 4B could be moved to a Supplementary Figure.

Answer: We modified the figure 4 according to the reviewer’s suggestion.

Okay

7. It’s unclear how data in Figure 4B, as cited, support the claim made in lines 309-310.

Answer: We corrected in the main text Line 421-422: “*Atoh1*-mediated SC-to-HC conversion in adult mouse cochleae shares similar patterns with normal SC-to-HC development.”

Please cite the data/figure panel in your manuscript that supports this statement.

8. In line 320, the authors state “These findings further solidify Atoh1-mediated regenerative approaches for hearing restoration…”. It is not clear at all how any of their results allows such a claim.

Answer: We modified the text Line 433-435 to “These findings further suggest that additional transcription factors or regulons need to be manipulated simultaneously or sequentially to ensure the proper and efficient conversion taking place.”

This reviewer agrees the point being made in this revised sentence addresses the issue raised previously. However, the sentence could be worded better.

9. Although the authors have mostly done a decent job of describing the rationale behind the work and their results, there is clearly some room for improvement in writing. This ranges from typos (e.g., line 319), possible mistakes (e.g., “insignificant” in line 266) to unsupported claims (e.g., “…acting as master regulators…” in lines 90-91 where the wording should’ve been “…may act as…”), unintelligible framing (e.g. “…Based on VIPER, the with the top 20 MRs found in the cHC3: OHC_P7 comparative analysis was presented in Figure 4A.” in line 258) and inappropriate content structure (e.g., lines 258-273 contain text written almost like figure legend, not results).

Answer: We improved the writing by correcting those.

Line 270 of the revised manuscript still reads “Based on VIPER, the with the top…” [same error as before]
Line 271 reads “Atoh1 shows as the most significant candidate… “ [new sentence has poor construction]
Line 272 reads “The color panels on the right represented…” [this still sounds like a figure legend]

Based on these examples, this reviewer disagrees that the authors have corrected the issues raised previously regarding wording and sentence structures. There are still numerous examples of poorly structured sentences, typos, grammatical errors throughout the manuscript, not just in the examples cited here.
