# Supplementary material for: Systematic single cell RNA sequencing analysis reveals unique transcriptional regulatory networks of Atoh1-mediated hair cell conversion in adult mouse cochleae
Source: PLoS One. 2023 Dec 11;18(12):e0284685. doi: 10.1371/journal.pone.0284685 (PMC10712870; doi:10.1371/journal.pone.0284685)
Supplement: Attachment — Submitted filename: Response to editor.docx [file pone.0284685.s009.docx]

1. One of the major concerns of Reviewer 1 and the Editor is the methods, in particular the integration analysis. This can be clarified if all code is easily followed and shared on GitHub.

Author’s response: The github page is re-organised and here is the new link: [*https://github.com/shutu0302/sc-paper*](https://github.com/shutu0302/sc-paper). Pre-processed data files are also uploaded to <https://drive.google.com/drive/folders/1RYiemRLYgBm2D8WaYobz3GTPgZGnVo8C?usp=share_link>. The link is also accessible thru github page.

2. The following needs to be clarified (as included in Reviewer 1's comments):

- Lines 116-118: Figure 1C - add DEG between IHCs vs OHCs. Can be done using scanpy or a wilcoxon rank sum test with the sctransform counts.

Author’s response: Figure of DEG comparing IHCs vs OHCs is done using scanpy and is added as Figure 1D.

- Line 129: reference Figure 2A at the end of this sentence.

Author’s response: Figure 2A is referenced in this sentence as suggested: “In this study, we aimed to identify cell type specific GRNs for the cells involved in the conversion process **(Figure 2A)**.” (Line 110)

- Lines 204-206: Interpretation of Fig 3A. This is not a "bifurcation" per se, but multiple lineages as there are 4 branches. Further, state limitation of this type of analysis as there's one lineage (from LPsC through OHC_P1 and to OHC_P7) that has arrows pointing in both directions (in the middle of OHC_P1), unless that's a separate lineage within the OHC_P1s as the circle dot is not actually on the main lineage. In either case, please clarify language.

Author’s response: We modified and clarifies this to “Unlike Monocle analysis2, PAGA TI generated distinct branches from LPsCs (Figure 3A). LPsCs committed to HCs follow a trajectory path from LPsCs to iHC_E14 and iOHC_E16, and finally into OHC at P1 and P7. Meanwhile, other lineages of LPsCs give rise to supporting cells like IPC or OPC. Nevertheless, we still observed some uncertainties in the trajectory, such as the backward arrow pointing from OHC_P1 to iOHC_E16. PAGA assumes that the trajectory is a tree-like structure with clear directionality, but some biological processes may not follow a strict linear progression. This can lead to inaccuracies in the analysis of certain cell types or developmental stages.” (Line 171-178)

- Lines 236-237: Should reference Fig S4A and B, not S3A and B.

Author’s response: Thank you for your suggestion. We figured that it is not necessary to perform trajectory analysis on the combined dataset. We now understand that the conversion path from SC to cHCs is different from natural hair cell development and including cells from different developmental stages and experimental conditions could cause confusion. Therefore, we have decided to remove Figure S4A-B and instead focus on our main findings. We have also renamed the original Figure S5 as Figure S4 to maintain the numbering consistency. We hope that these changes will improve the clarity of our manuscript.

- Include a statement in the discussion regarding the difficulty of batch effects and integration of datasets from multiple platforms.

Author’s response: We included a statement in our discussion “Integrating scRNA-seq data from multiple platforms can be challenging due to differences in library preparation, sequencing depth, and experimental conditions, leading to batch effects that can obscure biological signal and confound downstream analysis. We found SCTransform can provide improved expression correlation results.” (Line 221-225)

3. Please see comments from Reviewer 2 that are directly on the attached PDF (in blue text).

Author’s response: The response to Reviewer 2 is in the word file “Response to reviewer 2”.

4. There are a number of grammatical issues that I've identified and detailed below for correction.

Author’s response:

Grammar/style:

31: "albeit broad interest" to "albeit of broad interest" - corrected

53: take out "the" in front of "the target gene expression"

- We modified that sentence to “The development of sensory hair cells (HCs) in the inner ear is regulated by a complex gene regulatory network (GRN) involving transcription factors and their associated cofactors.” (Line 55)

56: take out "a" in front of "a key to"

- We restructured that sentence.

70: "regenerating" should be "regeneration"

- We restructured that sentence to: “Efforts to regenerate HC-like cells in adult mouse cochleae in vivo have had limited success, with the newly converted HCs (cHCs) from SCs remaining immature and non-functional.” (Line 60-61)

71: take out "the state-of-the-art"

- corrected by reconstructing the sentence

85: "change" should be "changes"

- corrected by reconstructing the sentence

89: "suggested" should be "suggest"

- we correct this by reconstructing the sentence to “Moreover, we identified key regulators, including Zbtb20, Nfia, Zmiz1, Gm14418, Bhlhe40, Six2, Fosb and Klf9, as candidate regulators that may promote the regeneration of more functional HCs in adult mammals.” (Line 79-81)

91: "provided" should be "provides"

- corrected by reconstructing the sentence to “Our study serves as an example of how to perform in-depth analysis with large scRNA-seq datasets and provides important insights into the regulatory dynamics of cochlear HC regeneration, which could guide future experimental approaches to hearing restoration in vivo.” (Line 81-83)

91: "of how" not "as how"

- corrected by reconstructing the sentence to “Our study serves as an example of how to perform in-depth analysis with large scRNA-seq datasets and provides important insights into the regulatory dynamics of cochlear HC regeneration, which could guide future experimental approaches to hearing restoration in vivo.” (Line 81-83)

108: should read "the Scanpy platform" (insertion of "the")

- corrected by reconstructing the paragraph.

126-127: remove "the following"

- corrected by reconstructing the paragraph. The sentence is reconstructed to “However, we did not perform co-expression network analysis or more specific GRN analysis.” (Line 108-109)

141: should be "Figure S1A"

- corrected; and all the figure reference formats were checked and corrected.

145: should this be "Supplementary Table 1" or just "Table 1"?

- corrected as “Supplementary Table 1”

164: should be "Supplementary Table 1"?

- Should be “Table 1” we have in the manuscript (Line 497). To avoid confusion, we also uploaded a new Figure named “Table 1”.

173: instead of "invented", how about "developed"

- corrected in Line 144

196: change to "The previous study", instead of "previous study"

- corrected by reconstructing the sentence to “In the past, we identified cHC3 as the primary cluster of highly differentiated cHCs in P33 cochleae, which had a resemblance to differentiating neonatal HCs.” (Line 165-167)

204: shouldn't this be "the bifurcation of development" instead of "the development"?

- corrected by simply mentioning “Unlike Monocle analysis, ……” (Line 171)

222: take out "the" infant of "batch effect"

- corrected by reconstructing the sentence to “OHC_P1 formed two subsets as it was prepared in two batches in the original paper” (Line 184-185)

222-224: combine and modify these two sentences:

"Given that distances between clusters may not be meaningful in

2-d as it uses local notions of distance to construct its high-dimensional graph representation, we also

analyzed the heatmap of the top 10 DEGs for each cluster."

- Corrected by restructuring the sentence to “The heatmap for the top 10 DEGs in each cluster revealed that cHCs3 expressed cell markers of OHC_P1 alongside an additional panel of genes (Figure 3D).” (Line 185-186)

226: take out "the" in front of "natural HC"

- Corrected by restructuring the sentence to “The heatmap for the top 10 DEGs in each cluster revealed that cHCs3 expressed cell markers of OHC_P1 alongside an additional panel of genes (Figure 3D).” (Line 185-186)

228: take out "also" after "We"

- Corrected by mentioning “….alongside an additional panel of genes (Figure 3D).” (Line 185-186)

232: typo/misspelling "previous" instead of "pervious"

- The sentence is moved to Line 243, and the typo is corrected there.

237: change "nature" to "natural"

- We removed this sentence after taking out original figure S4.

248: remove "the with"

- Corrected by reconstructing the sentence to “Figure 4 displays the top 10 regulators identified by VIPER in the cHC3: OHC_P71; 2 comparative analysis.” (Line 204-205)

249: "is" instead of "was"

- Corrected (Line 205)

250: "there are" should be "there is"

- Corrected to “…given the ectopic Atoh1 expression in cHCs.” (Line 205-206)

250: "represent" instead of "represented"

- Corrected by reconstructing the sentence to “The right-side color panels show the expression value and estimated protein activity of each transcription factor in the dataset.” (Line 206-207)

252: "provide" instead of "provided"

- Corrected by reconstructing the sentence to “These parameters indicate the significance of these genes in shaping the characteristics of either cHC3 or OHC_P7.” (Line 207-208)

298-299: instead of "the naturally HC development" either "natural HC development" or "the naturally developing HC"

- Corrected (Line 244)

304: "natural" instead of "nature"

- Corrected by reconstructing the paragraph.

315: change to: has been considered a "pro-hair cell gene" (replacing as with "a" and taking out "s")

- Corrected (Line 254)

358: remove "." at the end of single-cell data and replace with "("

- Corrected (Line 297)

647: shouldn't this be "correlated with" instead of "cooperated with"?

- We removed Figure S4

Figure 4 legend: should read "Darker values represent higher values" instead of "Darker the color, higher the value"

- Corrected (Line 540)

Note: make all figure references the same throughout the manuscript. An example of inconsistent: "S1 Figure A" (line 141) and "S5 Fig. A" (line 255). Please comb through the manuscript for these.

- Corrected all figure reference errors.
